# Castor oil and europium-based luminescent films for thermal sensing

**DOI:** 10.3389/fchem.2026.1788881

**Published:** 2026-02-23

**Authors:** Rodolpho A. N. Silva, Fernando E. Maturi, Gabriel L. Colombo, Bruno S. D. Onishi, Beatriz D. Freitas, Fábio J. Caixeta, Marian R. Davolos, Marco A. Cebim, Luís D. Carlos, Sidney J. L. Ribeiro, Sergio A. M. Lima, Ana M. Pires

**Affiliations:** 1 Department for Sustainable Development and Ecological Transition, Università del Piemonte Orientale “A. Avogadro”, Vercelli, Italy; 2 Institute of Chemistry, São Paulo State University (Unesp), Araraquara, Brazil; 3 Phantom-g, CICECO - Aveiro Institute of Materials, Department of Physics, University of Aveiro, Aveiro, Portugal; 4 Nanomaterials for Bioimaging Group, Departamento de Física de Materiales, Facultad de Ciencias, Universidad Autónoma de Madrid, Madrid, Spain; 5 São Carlos Institute of Chemistry, University of São Paulo (USP), São Carlos, São Paulo, Brazil; 6 Department of Chemistry, Federal University of São Carlos (UFSCar), São Carlos, São Paulo, Brazil; 7 School of Technology and Sciences, São Paulo State University (Unesp), Presidente Prudente, São Paulo, Brazil

**Keywords:** films, lanthanides, luminescence, sensors, temperature, thermometry

## Abstract

Luminescent thermometry has emerged as a powerful tool for remote temperature sensing, yet the development of sustainable materials that combine robust photophysical performance with environmental compatibility remains a challenge. Herein, we report a bio-derived luminescent thermometric film obtained by incorporating the europium-based complex [Eu (tta)_3_(PIB)] into a castor-oil-based alkoxysilane polymer (SiCO). The resulting luminescent films are transparent, stable, and preserve the structural integrity and optical characteristics of the trivalent europium (Eu^3+^) complex, as confirmed by spectroscopic analyses. Efficient ligand-to-metal energy transfer gives rise to well-defined Eu^3+^ emission, while residual ligand-centered luminescence enables a ratiometric thermometric approach. Temperature-dependent photoluminescence measurements reveal distinct thermal quenching behaviors of the ligand and Eu^3+^ emissions, allowing reliable temperature readout through an intensity ratio thermometric parameter. The optimized SiCO-0.25Eu film exhibits a maximum relative thermal sensitivity of 1.31% K^−1^ at 189 K and a minimum temperature uncertainty of 0.43 K at 173 K, maintaining stable performance over a broad low-temperature range (42–282 K) and under repeated thermal cycling. These results demonstrate that castor-oil-derived polymer matrices can serve as efficient and sustainable platforms for luminescent thermometry, offering a promising route toward environmentally friendly luminescent temperature sensors for low-temperature applications.

## 1 Introduction

In recent decades, substantial attention has been devoted to the development of luminescent materials that combine high efficiency, low production costs, straightforward synthesis at both laboratory and industrial scales, and enhanced environmental compatibility. Among these materials, lanthanide-containing compounds stand out because of their unique optical properties, which enable their integration into a wide range of systems, including nanoparticles, polymers, inorganic matrices, and coordination complexes (Alexander et al., 2025; Liu et al., 2024; Sivakumar and Lee, 2024).

Lanthanide-based nanoparticles are often regarded as the preferred option, as inorganic host matrices provide excellent photostability. However, their translation into real-world applications is frequently hindered by significant batch-to-batch variability and the limited availability of long-term cytotoxicity studies (DaCosta et al., 2014; Yang et al., 2024). Lanthanide coordination compounds, on the other hand, offer structural versatility, allowing precise tuning of their luminescent properties through rational modification of the ligands coordinated to the metal center (Bryleva et al., 2025; Hasegawa et al., 2018; Shavaleev et al., 2015). This tunability has enabled applications in optical devices, such as LEDs and OLEDs, as well as in sensing and bioimaging (Liu et al., 2024; Sivakumar and Lee, 2024). Despite these advantages, many lanthanide coordination complexes suffer from limited photostability under ultraviolet (UV) excitation, which remains a significant obstacle to their widespread practical use (Kai et al., 2011; Wei et al., 2016).

To overcome this limitation, several strategies have been explored, among which the incorporation of luminescent complexes into polymeric matrices has proven particularly effective (Ilmi et al., 2019). This approach enables the fabrication of homogeneous films with high emission intensity and enhanced photostability (Ilmi et al., 2019). Poly (methyl methacrylate) (PMMA) is one of the most widely used matrices due to its low cost and stabilizing effect on luminescent systems (Assunção et al., 2025). However, PMMA processing commonly relies on chlorinated solvents, such as dichloromethane and chloroform, raising concerns related to environmental sustainability (Essahili et al., 2024). As a result, growing efforts have been directed toward identifying alternative polymeric matrices that reduce ecological impact while maintaining, or even improving, luminescent performance.

In this context, silicon-based polymers have emerged as attractive candidates for luminescent film fabrication due to their high thermal stability, mechanical flexibility, and compatibility with luminescent species (Fang et al., 2021). Notably, Simões et al. reported polysiloxane-based films incorporating a trivalent europium ion (Eu^3+^) complex for white-light emission, where the characteristic red emission of Eu^3+^ was combined with the blue-green emission of the polymer matrix to yield balanced white light (Simões et al., 2018). More recently, other environmentally friendly matrices, including cellulose derivatives, urethanes, and silicon-based materials, have been explored for luminescent applications (Arauzo et al., 2025; Gao et al., 2023; Silva et al., 2024a). Although castor oil (CO, primarily composed of ricinoleic acid) is among the most extensively investigated renewable resources for polymer synthesis (Pinto et al., 2015) and can improve the thermal stability of Eu^3+^ complexes (Caixeta et al., 2025), its potential in luminescent applications remains underexplored, highlighting both the relevance and novelty of the present work.

Beyond optical devices and sensing, luminescent materials are also desirable for luminescence thermometry, which exploits the temperature dependence of their emission properties to enable remote temperature readouts (Brites et al., 2019; Dramićanin, 2020). In this context, Eu^3+^ complexes containing β-diketonate ligands are particularly appealing due to their intense red emission, pronounced thermal sensitivity, and enhanced stability when embedded in polymeric matrices. Therefore, in this study, the Eu^3+^ complex containing the ligands 4,4,4-trifluoro-1-(thiophen-2-yl)butane-1,3-dione (tta) and 2-phenylimidazo(4,5-f) (1,10) phenanthroline (PIB), [Eu (tta)_3_(PIB)], was incorporated into an alkoxysilane-castor oil-based polymer (SiCO), an environmentally friendly matrix derived from CO, at concentrations ranging from 0.25 to 3.00 wt%. The luminescent properties of the resulting composite films were systematically investigated, with special emphasis on the thermometric performance across the 13–312 K temperature range.

## 2 Materials and methods

### 2.1 Materials

Europium oxide (Sigma, 99.99%), 4,4,4-trifluoro-1-(thiophen-2-yl)butane-1,3-dione (tta, Sigma, ≥98%), acetanilide (Sigma, 99%), sodium hydroxide (NaOH, Neon, ≥98%), hydrochloric acid (HCl, Sigma, 37%), ethanol (Neon, ≥99%), methanol (Neon, ≥99%), hexane (Neon, ≥99%), tetrahydrofuran (THF, Neon, ≥99%), 3-(Triethoxysilyl)propyl isocyanate (ICPTES, Aber, 95%), castor oil (VWR Chemicals, GPR grade), and chloroform (Neon, ≥99%) were purchased and used without further purification. All the following syntheses were carried out under standard atmospheric conditions.

### 2.2 Synthesis of europium-based complex

The ligand 2-phenylimidazo(4,5-f) (1,10)phenanthroline (PIB) was first synthesized following a procedure described elsewhere (Li et al., 2014) and then used with the ligand tta to obtain the luminescent [Eu (tta)_3_(PIB)] complex. Briefly, 0.15 g of tta and 0.07 g of PIB (3:1 M ratio) were added to a beaker containing 10 mL of methanol and 5 mL of THF. A methanolic NaOH solution was added dropwise until the pH reached 6. The resulting mixture was stirred at 50 °C for 30 min and subsequently transferred to a reaction flask containing 2.1 mL of a 0.1 mol L^−1^ europium chloride solution (prepared by acid dissolution of europium oxide in HCl). The reaction mixture was then refluxed at 85 °C for 4 h. After completion, the solution was cooled to room temperature, and the volatile components were removed, yielding an orange solid. The crude product was dissolved in chloroform and reprecipitated with hexane, followed by filtration and washing four times with 10 mL portions of cold hexane. The final product was dried in a desiccator, resulting in a pale-yellow solid with a yield of 70%. The complex was characterized as described in the reference (Silva et al., 2024b). Elemental analysis of [Eu (tta)_3_(PIB)] (molecular weight = 1,111.82 g mol^−1^) gave the following values (found/calculated): C, 46.55% (46.45%); H, 2.18% (2.18%); N, 5.64% (5.04%). Standard acetanilide (found/calculated): C, 71.13% (71.09%); H, 6.42% (6.71%); N, 11.37% (10.36%).

### 2.3 Preparation of the polymer precursor

The polymer precursor was synthesized following the methodology reported in (de Freitas et al., 2023). To prepare the SiCO precursor, castor oil was mixed with ICPTES in a 1:3 M ratio and stirred at 82 °C for 24 h. After synthesis, the SiCO precursor was stored in a plastic container at 10 °C for further use.

### 2.4 Fabrication of films

For the fabrication of the SiCO polymeric film, 0.750 g of the SiCO precursor was mixed with 5 mL of ethanol and 0.1 mL of 0.1 mol L^−1^ HCl in a beaker. The mixture was stirred for 2 h, then cast onto a plastic Petri dish using a drop-casting procedure. The film was dried at 40 °C for 48 h in a ventilated oven. To prepare the films containing the [Eu (tta)_3_(PIB)] complex, the same procedure was followed, adding 1.9 mg of the complex to 0.748 g of SiCO to obtain a concentration of 0.25% by weight. A pristine SiCO film and complex-containing films at 0.25, 0.50, 1.00, and 3.00% by weight were obtained and labeled as SiCO, SiCO-0.25Eu, SiCO-0.50Eu, SiCO-1.00Eu, and SiCO-3.00Eu, respectively.

### 2.5 Characterization

Fourier transform infrared vibrational (FTIR) spectra were collected on a Vertex 70 spectrometer (Bruker) equipped with a DLATGS detector, acquiring 64 scans at 1 cm^−1^ resolution over the 400–4,000 cm^-1^ range using a diamond attenuated total reflection (ATR) crystal. Elemental CHN analyses were obtained using a FlashEA 1,112 analyzer (Thermo Scientific) calibrated with BBOT, sulfanilamide, and cystine standards at 950 °C. Ultraviolet–visible (UV-Vis) absorption spectra of ethanolic solutions (1 μmol L^−1^) were recorded at room temperature using a Cary 60 spectrophotometer (Agilent Technologies). Transmittance spectra were collected at 298 K with a Lambda 950 dual-beam spectrophotometer (PerkinElmer) over 250–800 nm with a 1.0 nm resolution. Photoluminescence spectra of the SiCO-0.25Eu film in the solid state were measured between 13 and 312 K using a Fluorolog-3 FL3-122 spectrofluorometer (Horiba Jobin Yvon) equipped with a R928P photomultiplier (Hamamatsu) and a 450 W Xenon short-arc lamp (UXL-450S-O, Ushio Inc.) for steady-state excitation. Emission intensity decay profiles were recorded in the same equipment using a 150 W Xenon flash lamp (FX-1102, Excelitas Technologies) with 0.15 J per flash. The obtained decay curves were adjusted to a monoexponential decay function $I\left(t\right)=I_{0}+A\exp\left(-t/\tau \right)$ , where *I*(*t*) is the intensity as a function of the time *t*, *I*_0_ is the intensity background, is the intensity amplitude, and *τ* is the emission lifetime (Thor et al., 2024). Although it is common practice to use the standard error of the fitted value as the uncertainty of *τ*, the resulting values are lower than the actual temporal resolution of the equipment. In this sense, we have used herein the time increment (0.05 m) as the uncertainty of *τ* to better reflect the equipment’s temporal accuracy. Temperature-dependent photoluminescence spectra and decay curves were measured in a closed-cycle helium cryostat under vacuum (4 × 10^−4^ Pa), controlled by a Lakeshore 331 temperature controller and monitored with a DT-470-SD silicon diode sensor, with accuracies of ±0.5 K (12–30 K), ±0.25 K (30–60 K), and ±0.15 K (60–340 K).

### 2.6 Thermometric analysis

The relationship between temperature and the luminescent behavior of the SiCO-0.25Eu film was determined by establishing an intensity ratio between the integrated emission bands from the PIB ligand (*I*_PIB_, 400–575 nm) and Eu^3+^ ^5^D_0_→^7^F_2_ transition (*I*_Eu_, 604–640 nm) taken from the emission spectra at each temperature, resulting in the thermometric parameter Δ = *I*_PIB_/*I*_Eu_. After the baseline subtraction, all spectra were converted from wavelength to energy (cm^−1^) using the Jacobian transformation (Mooney and Kambhampati, 2013), where the intensity ratio as a function of the temperature was adjusted to a Boltzmann-type sigmoidal function. The relative thermal sensitivity $S_{r}=\frac{1}{\Delta }\left|\frac{d\Delta }{dT}\right|$ was used to quantify the performance of the thermometer’s responsiveness to temperature variations, where dΔ/dT indicates the first derivative of Δ (Brites et al., 2023). The uncertainty in temperature $\delta T=\frac{1}{S_{r}}\frac{\delta \Delta }{\Delta }$ represents the smallest temperature change detectable by Δ. The uncertainties of the integrated emission intensities *I*_PIB_ and *I*_Eu_ were determined by propagating the baseline noise through the numerical integration procedure. The noise amplitude was estimated as the root-mean-square fluctuation of the Jacobian-corrected intensity in the 714–735 nm spectral region (where there is no emission from the ligand nor from Eu^3+^). Because the trapezoidal integration corresponds to a weighted linear sum of the spectral intensities, the variance of the integrated area was calculated as the sum of the squared trapezoidal weights multiplied by the noise variance, explicitly accounting for the non-uniform energy spacing of the energy-converted spectra. The resulting standard deviation represents the statistical uncertainty associated with each integrated emission intensity. The uncertainty in Δ (δΔ) was then calculated by propagating the uncertainties of the integrated areas.

## 3 Results and discussions

### 3.1 Molecular and optical characterization

**FIGURE 1:**
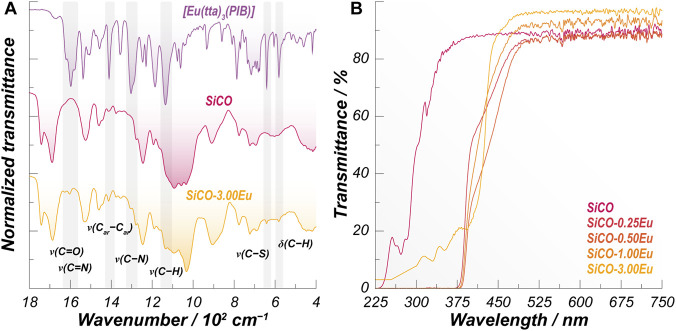
**(A)** ATR-FTIR spectra of the complex [Eu (tta)_3_(PIB)], pure SiCO, and SiCO-3.00Eu films. **(B)** UV-Vis transmittance spectra of the pristine and complex containing SiCO films.

To investigate the molecular-level interactions between the complex and the polymer matrix, FTIR spectra were collected for the [Eu (tta)_3_(PIB)] complex, the pristine SiCO film, and the SiCO-3.00Eu film (Figure 1A). The film with a higher complex concentration was intentionally selected to resolve its characteristic vibrational bands more clearly (Rozhkov et al., 2013; Silverstein et al., 2005), which tend to overlap with SiCO vibrational modes at lower concentration. The distinct features of the complex remain detectable in the composite film without noticeable shifts, indicating that its structural integrity is preserved within the polymer network. The full FTIR spectrum of the [Eu (tta)_3_(PIB)] complex and the assignment of the vibrational modes can be seen in Supplementary Figure S1 and Supplementary Table S1, in the Supplementary Material.

Complementary optical characterization was performed on the [Eu (tta)_3_(PIB)]-containing SiCO films, as shown in Figure 1B. The pure SiCO film displays two main absorptions at 272 and 320 nm (Figure 1B), corresponding to the π→π^*^ and *n*→π^*^, respectively, from the CO chains and the C=O and C=N groups within the SiCO structure (de Freitas et al., 2023). The complex-containing films exhibit enhanced absorption in the 275–450 nm region relative to pure SiCO (Figure 1B), which accounts for the characteristic absorptions from the SiCO host matrix plus the absorption coming from the π→π^*^ transition in the tta and PIB ligands (Arauzo et al., 2025; Josephine Kanimozhi and Alexander, 2017). A weak absorption tail in the 380–450 nm range, responsible for the film’s faint yellowish hue, is attributed to ligand-centered transitions in the coordinated complex or to partial π–π stacking interactions involving aromatic rings (Mutti et al., 2021). Such interactions subtly alter the electronic environment of the ligands, producing small spectral shifts while preserving the overall optical response of the material. In the end, the obtained films present a transparency higher than 85% in the 450–750 nm range.

### 3.2 Photoluminescent response

**FIGURE 2:**
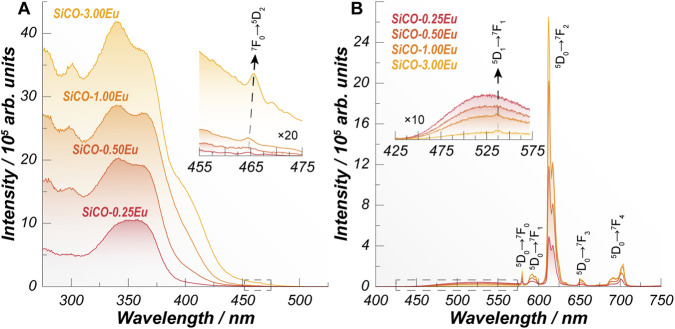
**(A)** Excitation spectra monitoring the emission at 612 nm and **(B)** emission spectra under excitation at 254 nm of the different films containing the [Eu (tta)_3_(PIB)] complex at different concentrations. The insets in panels **(A,B)** display the zoomed intensities of the dashed regions.

The excitation spectrum of the [Eu (tta)_3_(PIB)]-containing SiCO films, monitored at the ^5^D_0_→^7^F_2_ transition of Eu^3+^ (Figure 2A) exhibits broad bands assigned to the *S*_n_←*S*_0_ transitions of the tta and PIB ligands (Arauzo et al., 2025). The low intensity of sharp f–f lines (or absence, for samples SiCO-0.25Eu and SiCO-0.50Eu), characteristic of direct Eu^3+^ excitation, indicates that the population of the ^5^D_0_ level occurs predominantly through ligand absorption followed by ligand-to-metal energy transfer via the antenna effect (Binnemans, 2015). A weak feature near 400 nm is also observed and is attributed to π–π stacking interactions, consistent with tail absorption seen in the transmittance spectra (Rozhkov et al., 2013).

Upon 254 nm excitation, the emission spectra of the films (Figure 2B) show the characteristic narrow bands of Eu^3+^ related to the ^5^D_0_→^7^F_0–4_ set of transitions superimposed on a broad band attributed to emissions from the SiCO matrix and the coordinated ligands (de Freitas et al., 2023; Gao et al., 2017). Derivatives of 1,10-phenanthroline, as the PIB ligand, are known to display intense visible fluorescence, with peak positions dependent on their molecular framework (Bing et al., 2004; Josephine Kanimozhi and Alexander, 2017). Although such a band is absent in the powdered complex, incorporation into the polymer matrix modifies the local chemical environment and the orbital overlap within the ligand system. These effects can reduce the efficiency of ligand-to-metal energy transfer, resulting in the residual broad band ligand-centered emission observed in the 425–575 nm region (Dandekar et al., 2018; Yang et al., 2016).

To gain further insight into the Eu^3+^ local environment, the Judd-Ofelt intensity parameters Ω_2_ and Ω_4_ were calculated for the complex in both powdered form and when embedded in the SiCO matrix, as presented in Table 1. The Ω_2_ parameter is commonly associated with the degree of asymmetry and covalency at the coordination site, reflecting the polarizability of the Eu^3+^ surroundings, whereas Ω_4_ is more sensitive to the rigidity and vibrational properties of the host, as well as to subtle changes in Eu–ligand bonding (Bünzli, 2015; Silverstein et al., 2005). Upon incorporation into the polymer matrix, both parameters exhibit variations, evidencing a clear modification of the Eu^3+^ coordination environment. In particular, the moderate increase in Ω_2_ indicates that the metal center experiences a more distorted and polarizable local field, consistent with a reduced symmetry imposed by the SiCO polymer framework. In contrast, the more pronounced enhancement of Ω_4_ suggests a strengthening of the ligand field and a concomitant increase in environmental rigidity, likely arising from constrained Eu–ligand vibrational modes and subtle reorganization of coordination interactions induced by the SiCO matrix. This indicates that, despite the molecular integrity of the [Eu (tta)_3_(PIB)] complex being preserved after incorporation into the SiCO polymer, as shown by the FTIR results (Figure 2A), the polymeric surrounding environment plays a role in the complex coordination interactions.

**TABLE 1:** Judd-Ofelt parameters of the [Eu (tta)_3_(PIB)] complex in the solid state and after incorporation into the SiCO matrix, highlighting the variations induced by the host environment.

Sample	Ω_2_/10^–20^ cm^2^	Ω_4_/10^–20^ cm^2^
[Eu (tta)_3_(PIB)]^a^	26.07	5.96
SiCO-0.25Eu	24.70	8.17
SiCO-0.50Eu	24.43	8.13
SiCO-1.00Eu	26.02	8.45
SiCO-3.00Eu	25.27	8.60

^*a*^ From reference (Silva et al., 2024b).

### 3.3 Temperature-dependent behavior

**FIGURE 3:**
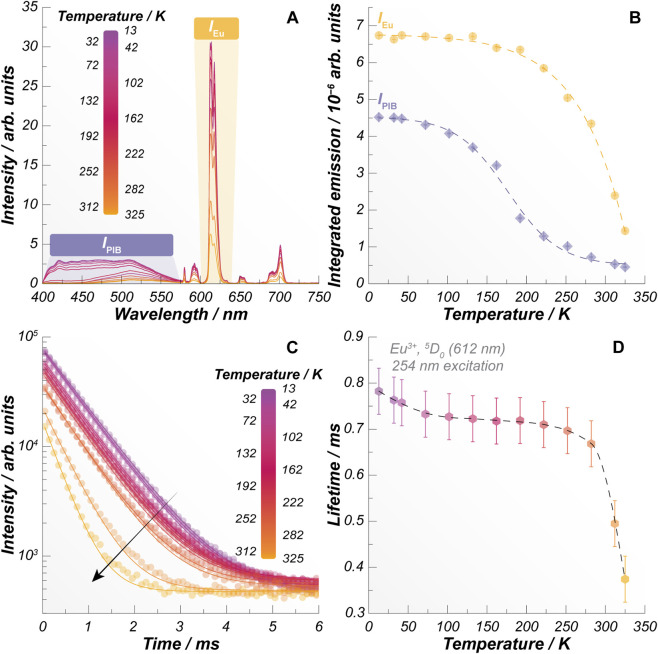
**(A)** Temperature-dependent emission of the SiCO-0.25Eu film under 254 nm excitation. **(B)** Thermal evolution of the integrated emissions *I*_PIB_ and *I*_Eu_, calculated after the Jacobian transformation of the highlighted regions in panel **(A). (C)** Emission decay profiles of Eu^3+^ ^5^D_0_ emitting level monitored at 612 nm under 254 nm excitation and **(D)** the corresponding emission lifetimes. Dashed lines in **(B)** and **(D)** are guides for the eyes, while the solid lines in **(C)** are the monoexponential decay fits (*r* ^2^ > 0.998).

Among the different films incorporating the [Eu (tta)_3_(PIB)] complex, the SiCO-0.25Eu sample exhibits the highest relative contribution from the PIB-centered emission. Importantly, this formulation provides well-resolved emission from both the ligand and Eu^3+^ while requiring the lowest complex loading, making it an optimal platform to investigate temperature-dependent luminescence. Consequently, this sample was selected for thermal studies over the 13–312 K range (Figure 3A). As the temperature increases, the Eu^3+^ emission associated with the ^5^D_0_→^7^F_2_ transition (*I*_Eu_) undergoes an exponential quenching, while the ligand-centered emission (*I*_PIB_) displays a sigmoidal intensity decrease (Figure 3B). This contrasting behavior reflects the different deactivation pathways governing each emitting center. In particular, the strong thermal quenching of the Eu^3+^ emission is consistent with the progressive activation of non-radiative relaxation channels at higher temperatures. This interpretation is further supported by the systematic shortening of the Eu^3+^ ^5^D_0_ excited-state lifetime with increasing temperature (Figures 3C,D), which directly contributes to the overall emission intensity reduction of the material.

**FIGURE 4:**
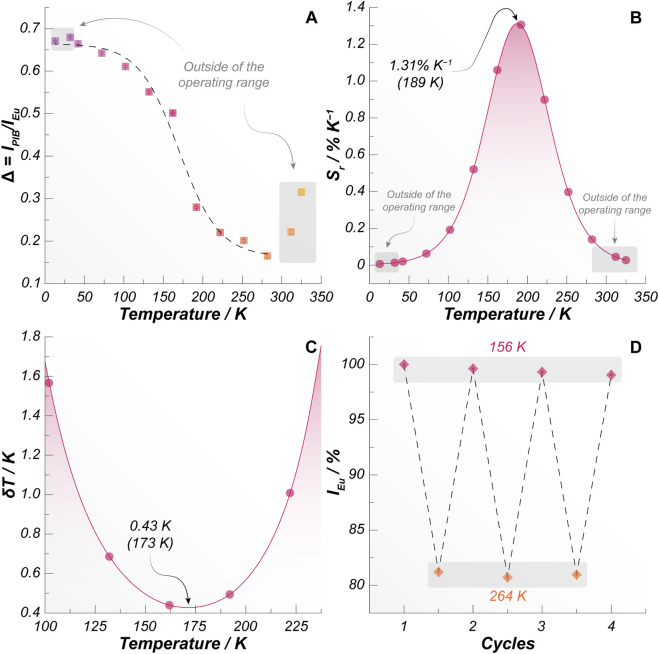
**(A)** Calibration curve of the intensity ratio as a function of the temperature. The solid line is the Boltzmann fit (*r* ^2^ > 0.986). **(B)** Relative thermal sensitivity and **(C)** uncertainty in temperature of the SiCO-0.25Eu film. The symbols correspond to the measured data, and the solid lines are the values calculated from the fitted data. Maximum *S*_r_ and minimum δ*T* values are indicated in panels **(B)** and **(C)**, respectively, as well as the corresponding temperature at which they take place. **(D)** Thermal stability of Eu^3+^ emission around the maximum *S*_r_ and minimum δ*T* temperature range during consecutive heating/cooling cycles.

Based on these complementary thermal responses, the ratio between the integrated areas of the ligand-centered emission band and the Eu^3+^ ^5^D_0_→^7^F_2_ emission was defined as the thermometric parameter Δ = *I*_PIB_/*I*_Eu_, indicating the relationship between the temperature-induced luminescent changes. The resulting calibration curve of Δ is shown in Figure 4A and reveals a well-defined, temperature-dependent luminescent response that follows a Boltzmann-like sigmoidal trend. It is important to note that above 282 K, *I*_Eu_ undergoes more pronounced thermal quenching than *I*_PIB_ (Figure 3B), leading to an inversion in the behavior of Δ, which begins to increase with increasing temperature. Under these conditions, the thermometric response becomes ambiguous, as a single Δ value may correspond to multiple temperatures. Similarly, the Δ values are pretty much the same between 13 and 42 K because *I*_Eu_ and *I*_PIB_ are nearly constant in this temperature range, as also observed in Figure 3B. As a result, the SiCO-0.25Eu sample works reliably and unambiguously as a luminescent thermometer within the 42–282 K temperature range.

Based on the temperature dependence of Δ, the relative thermal sensitivity *S*_r_ was calculated, reaching a maximum value of *S*_r_ = 1.31% K^−1^ at 189 K, as shown in Figure 4B. This sensitivity confirms that the system is well-suited for luminescence thermometry, as values equal to or exceeding 1.00% K^−1^ are generally regarded as adequate for practical sensing applications (Cheng et al., 2023; Gálico et al., 2019). Furthermore, the material maintains relatively high *S*_r_ values over a broad range of temperature, from 150 to 225 K, which highlights its robustness and applicability across an extended operational range rather than at a single optimal point. In addition to sensitivity, the temperature uncertainty (δ*T*) was evaluated to assess the precision of the thermometric readout, with the results summarized in Figure 4C. The minimum value of δ*T* = 0.43 K occurs at 173 K, indicating a high degree of accuracy in temperature determination within this regime. This low uncertainty, combined with the sensitivity across a wide temperature interval, further reinforces the suitability of the system for reliable luminescence-based thermometric investigations.

The stability of the SiCO-0.25Eu film was also assessed through thermal cycling experiments. After three consecutive heating-cooling cycles, no significant changes in the luminescent response were observed (Figure 4D). This result demonstrates that the [Eu (tta)_3_(PIB)] complex remains structurally and photophysically stable within the SiCO matrix and does not undergo detectable degradation upon repeated thermal stress. Consequently, the obtained film exhibits sufficient robustness for repeated use, preserving its thermometric performance over multiple cycles. For a broader perspective, Table 2 summarizes representative luminescent complexes embedded in polymer matrices reported in the literature, together with their corresponding relative thermal sensitivities and operational temperature ranges. This comparison places the present system within the current state of the art and highlights its competitive performance among polymer-based luminescent thermometers.

**TABLE 2:** Overview of luminescent thermometric systems embedded in polymer matrices and their operating temperature ranges, maximum relative thermal sensitivities, and minimum temperature uncertainties.

System	Matrix	Temperature range/K	Maximum *S*_r_/% K^−1^	Minimum δ*T*/K	Δ	Ref.
Eu^3+^ β-DKT	PLA film	298–353	11.7–20.1	–	Intensity/Lifetime	Knyazev et al. (2022)
Eu^3+^/Tb^3+^ β-DKT mix	Si-O-Si-based film	158–248	11.5	0.08	Eu/Tb intensity ratio	Gálico et al. (2018)
Tb^3+^ with carboxylic acid ligand	Coord. polymer	250–320	0.76	0.05	Ratiometric (intensity)	Costa et al. (2024)
Ce^3+^/Tb^3+^ MOF	Coord. polymer	313–473	1.37	0.36	Ratiometric (intensity)	Yue et al. (2023)
Tb^3+^/Eu^3+^ silsesquioxane	Si-O-Si-based film	273–373	0.63	0.04	Ratiometric (intensity)	Nigoghossian et al. (2021)
[Eu (tta)_3_(PIB)] complex	SiCO film	150–225	1.31	0.43	Ratiometric (intensity)	This work

## 4 Conclusion

In this study, a novel luminescent thermometric system was developed by incorporating the [Eu (tta)_3_(PIB)] complex into a castor-oil-derived polymer (SiCO), offering an environmentally friendly alternative to conventional synthetic matrices. Structural characterization by FTIR and optical transmittance spectroscopy confirmed that the coordination environment and molecular integrity of the complex were preserved upon incorporation into the polymeric network. The photoluminescent properties of the resulting composite films were thoroughly investigated, revealing efficient ligand-to-metal energy transfer and well-resolved Eu^3+^ emission. The suitability of the SiCO-based composite for luminescence thermometry was clearly demonstrated through the temperature-dependent luminescent response of the SiCO-0.25Eu film. The material exhibits a maximum relative thermal sensitivity (*S*_r_) of 1.3% K^−1^ at 189 K and achieves a minimum temperature uncertainty (δ*T*) of 0.43 K at 173 K, values that are fully competitive with polymer-embedded luminescent thermometers reported in the literature. Notably, the system maintains robust thermometric performance over a broad low-temperature range, highlighting its reliability operating beyond a single temperature.

Overall, these results establish the SiCO/[Eu (tta)_3_(PIB)] composite as a viable and reliable luminescent thermometer for low-temperature applications. By combining sustainable materials, structural and thermal stability, and competitive thermometric performance, this work provides a meaningful contribution to the development of more environmentally friendly luminescent sensing platforms and opens new avenues for the integration of bio-derived polymers into advanced optical thermometry.

## Data Availability

The raw data supporting the conclusions of this article will be made available by the authors, without undue reservation.
