# Supplementary material for: Castor oil and europium-based luminescent films for thermal sensing
Source: Front Chem. 2026 Feb 23;14:1788881. doi: 10.3389/fchem.2026.1788881 (PMC12968248; doi:10.3389/fchem.2026.1788881)
Supplement: Supplementary file 1 [file DataSheet1.pdf]

Supplementary Material

1 Supplementary Figures and Tables

1.1 Supplementary Figure

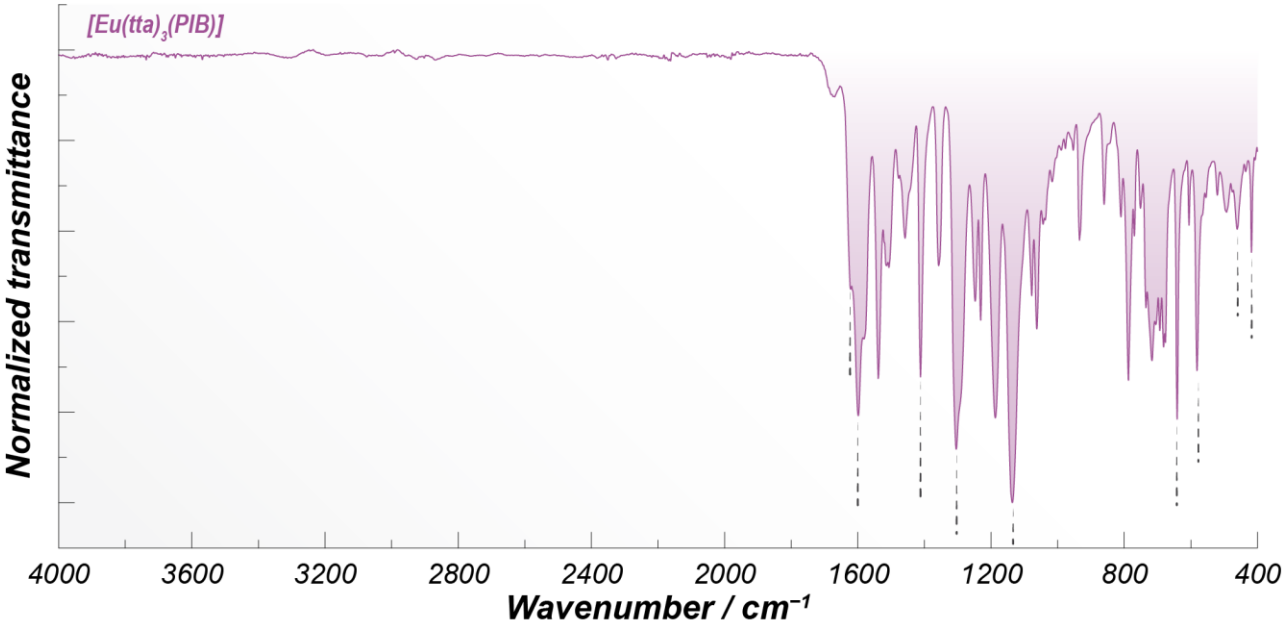

Supplementary Figure 1. ATR-FTIR spectrum of the $[Eu(tta)_3(PIB)]$ complex, with the dashed lines indicating the main vibrational modes.

1.2 Supplementary Table

Supplementary Table 1. Main vibrational modes observed in the ATR-FTIR spectrum of the $[Eu(tta)_3(PIB)]$ complex and their corresponding wavenumbers.

Vibrational mode	Wavenumber / cm^{-1}
OOP(C–H)[a]	416
ν(Eu–O)	460
δ(C–H)	580
ν(C–S)	641
ν(C–H)	1137
ν(C–N)	1304
ν(C_{ar}–C_{ar})	1410
ν(C=O)	1597
ν(C=N)	1623

[a]Out-of-plane ring bending of the PIB and the tta ligands.
